# Electronic Structure and Epitaxy of CdTe Shells on InSb Nanowires

**DOI:** 10.1002/advs.202105722

**Published:** 2022-02-18

**Authors:** Ghada Badawy, Bomin Zhang, Tomáš Rauch, Jamo Momand, Sebastian Koelling, Jason Jung, Sasa Gazibegovic, Oussama Moutanabbir, Bart J. Kooi, Silvana Botti, Marcel A. Verheijen, Sergey M. Frolov, Erik P. A. M. Bakkers

**Affiliations:** ^1^ Applied Physics Department Eindhoven University of Technology Eindhoven 5600 MB Netherlands; ^2^ Department of Physics and Astronomy University of Pittsburgh Pittsburgh PA 15260 USA; ^3^ Institut für Festkörpertheorie und ‐optik Friedrich‐Schiller‐Universität Jena Jena 07743 Germany; ^4^ Zernike Institute for Advanced Materials University of Groningen Groningen 9747 AG Netherlands; ^5^ Department of Engineering Physics Ecole Polytechnique de Montréal C.P. 6079, Succ. Centre‐Ville Montréal Québec H3C 3A7 Canada; ^6^ Eurofins Material Science Netherlands B.V. High Tech Campus 11 Eindhoven 5656 AE Netherlands

**Keywords:** CdTe, core–shell nanowires, heteroepitaxy, InSb, molecular beam epitaxy, density functional theory, transport

## Abstract

Indium antimonide (InSb) nanowires are used as building blocks for quantum devices because of their unique properties, that is, strong spin‐orbit interaction and large Landé g‐factor. Integrating InSb nanowires with other materials could potentially unfold novel devices with distinctive functionality. A prominent example is the combination of InSb nanowires with superconductors for the emerging topological particles research. Here, the combination of the II–VI cadmium telluride (CdTe) with the III–V InSb in the form of core–shell (InSb–CdTe) nanowires is investigated and potential applications based on the electronic structure of the InSb–CdTe interface and the epitaxy of CdTe on the InSb nanowires are explored. The electronic structure of the InSb–CdTe interface using density functional theory is determined and a type‐I band alignment is extracted with a small conduction band offset ( ⩽0.3 eV). These results indicate the potential application of these shells for surface passivation or as tunnel barriers in combination with superconductors. In terms of structural quality, it is demonstrated that the lattice‐matched CdTe can be grown epitaxially on the InSb nanowires without interfacial strain or defects. These shells do not introduce disorder to the InSb nanowires as indicated by the comparable field‐effect mobility measured for both uncapped and CdTe‐capped nanowires.

## Graphical abstract

Combining indium antimonide (InSb) nanowires with materials of dissimilar properties, has enabled the engineering of exotic materials, such as topological superconductors. This work explores potential applications of the material combination of InSb with cadmium telluride in the form of core–shell nanowire heterostructures. These heterostructures are studied in terms of growth, epitaxy, electronic structure of their interface, and electric transport properties.

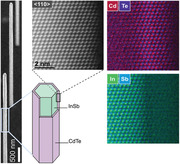

## 1 Introduction

Semiconductor nanowires with strong spin‐orbit coupling and large Landé g‐factor have unfolded novel research pathways in quantum transport ranging from spin phenomena^[^ ^1^, ^2^, ^3^, ^4^ ^]^ to quantum computing circuits,^[^ ^5^, ^6^, ^7^ ^]^ and most recently the pursuit of topological particles.^[^ ^8^, ^9^, ^10^ ^]^ Indium antimonide (InSb) is among these semiconductors, with the highest bulk electron mobility, largest Landé g‐factor, and strongest spin‐orbit coupling compared to other III–V materials.^[^ ^11^, ^12^ ^]^ Integrating these InSb nanowires with other materials holds great potential for realizing novel devices with unconventional functionality, yet it has been hampered in large part due to the large lattice constant of InSb that tends to complicate the realization of defect‐free and strain‐free heterostructures.

The II–VI material, cadmium telluride (CdTe), is an interesting material candidate as it nearly matches the large lattice constant of InSb with a lattice mismatch below 0.05% at room temperature. Moreover, their thermal expansion coefficients are comparable.^[^ ^13^ ^]^ Besides their structural compatibility, CdTe has a large bandgap compared to InSb. Therefore, combining InSb with CdTe is compelling for several device applications such as, quantum‐well lasers, high electron mobility transistors, and infrared detectors.^[^ ^14^, ^15^ ^]^ However, in spite of the nearly perfect lattice‐match, growth of CdTe‐InSb heterostructures remains complicated, due to preferential interface reactions which lead predominantly to the formation of an indium–tellurium rich interface region.^[^ ^16^ ^]^ The formation of such layer is undesirable, since different compositions of this indium telluride compound have different lattice constants and bandgaps.^[^ ^16^ ^]^ The ease of twin formation in CdTe crystals,^[^ ^17^, ^18^ ^]^ further complicates the realization of defect‐free interfaces.

Here, we combine InSb and CdTe in a core–shell (InSb–CdTe) configuration for potential applications based on the electronic structure of the InSb–CdTe interface, the structural quality, as well as, epitaxy of the CdTe shells on the InSb nanowires. The electronic structure of the InSb–CdTe is investigated with density functional theory (DFT) calculations, where both the bandgaps and the band‐edge alignment at the InSb–CdTe interface are extracted. In particular, we show that the electronic structure at the InSb–CdTe interface is well‐suited for passivating the InSb surface by virtue of a type‐I band alignment and for serving as a tunnel barrier when placed at the interface between the InSb nanowire and a metal or superconductor owing to a small conduction band offset of roughly 0.3 eV. In the context of engineering topological superconductors using superconducting–semiconducting nanowire hybrids, such a CdTe tunnel barrier could potentially minimize disorder and address the strong‐coupling issue between the superconductor and the nanowire. Disorder in these nanowire hybrids mimics the signatures of topological particles^[^ ^19^ ^]^ and an overly strong coupling is deemed to overwhelm the intrinsic properties of the semiconducting nanowire and possibly render topological superconductivity inaccessible.^[^ ^20^, ^21^ ^]^ Using CdTe shells at the interface between the nanowire and a superconductor with the extracted conduction band offset could modulate the superconductor–semiconductor coupling strength. In addition to the electronic structure of the InSb–CdTe interface, the crystal quality of the interface and of the CdTe shells is crucial for device applications. In particular, defected shells could induce strain in the InSb nanowire and introduce new sources of disorder that could impair the performance of the core–shell nanowires.^[^ ^22^, ^23^ ^]^ Therefore, we also demonstrate the growth of defect‐free, epitaxial CdTe shells with a smooth and abrupt interface to InSb nanowires. The growth parameters have been optimized to suppress interface reactions and therefore the formation of interface layers is suppressed entirely. Furthermore, the CdTe shell inhibits the InSb nanowire surface from oxidizing which eliminates the need for exposing the InSb nanowires to harsh chemicals to remove surface oxides for device fabrication. As discussed in Section 2.2, we determine that the CdTe shells are chemically stable against oxidation, where after a period of at least 3 weeks they remain oxide‐free. This property facilitates device fabrication, specifically in devices where the CdTe needs to be contacted, such as tunnel barrier devices. In this case, the metal or superconductor can be directly deposited on the CdTe without having to expose the CdTe to etchants and harsh chemicals. The quality of the grown shells is corroborated by transport measurements, where we obtain comparable electron mobility values for both bare, uncapped and CdTe‐capped InSb nanowires, thus confirming that these shells do not introduce additional disorder to the nanowires.

## 2 Results

### 2.1 Electronic Structure of the InSb–CdTe Interface

**Figure 1:**
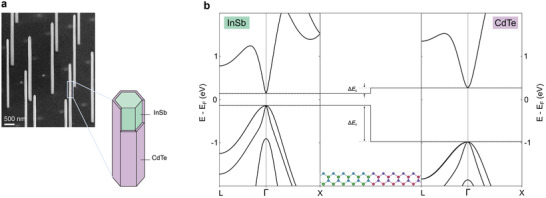
CdTe‐capped InSb nanowires and the electronic structure of the InSb–CdTe system. a) CdTe shells on InSb nanowires imaged at a 30°‐tilt with a scanning electron microscope (SEM). The schematic highlights the InSb core (green) surrounded by a CdTe shell (purple). For illustration purposes, the CdTe shell is removed on two facets to expose the InSb. b) On the left and right are the bulk bandstructures of InSb and CdTe, respectively, as obtained from density functional theory. In the central panel, the supercell composing the InSb–CdTe interface is shown with the band alignment. The potential difference between both materials is extracted from the averaged local electrostatic potential and is used for the alignment of both bandgaps at the interface. The band edge alignment has a conduction band offset Δ*E*_c_ = 0.15 eV and a valence band offset Δ*E*_v_ = −0.84 eV.

The electronic structure across the interface between the InSb and the CdTe is characterized using ab initio DFT calculations. The atomic structure of the interface (i.e., the supercell) along with selected results from the DFT calculations are graphically presented in **Figure** **1**b. The supercell, displayed in the middle panel of Figure 1b, is created based on experimental inputs extracted from the structural and composition analysis of the grown shells. Accordingly, the interface is oriented perpendicular to the <110 > crystal direction, in line with the CdTe coverage of the six equivalent {220} facets, which outline the hexagonal cross‐section of an InSb nanowire (Figure 1a). The supercell also accounts for the polarity of the InSb–CdTe interface, in accordance with the analysis discussed in Section 2, where Cd takes the position of In, and Te that of Sb. The nearly perfect lattice match allows for the assumption that both InSb and CdTe have the same lattice constant. This is substantiated by our observations of the absence of strain in the grown shells (see Section 2). Therefore, an ideal interface is assumed with the lattice constant of the supercell set to the experimental value of CdTe. Additional calculations, details, and results are provided in Section S1, Supporting Information.

The results presented in Figure 1b show the individual bulk bandgaps of both InSb and CdTe, as well as, the band alignment at the interface of the heterostructure, that is, the supercell. The potential difference at the interface, calculated from the local electrostatic potential of the supercell, is used to shift the InSb and CdTe bands, yielding the shown alignment. This alignment is commonly referred to as type‐I, with the InSb band‐edges lying between those of CdTe. From the band‐edge alignment a conduction band offset Δ*E*_c_ = 0.15 eV and valence band offset Δ*E*_v_ = −0.84 eV are extracted. Since the bandgap of CdTe is slightly underestimated–compared to experimental values—Δ*E*_c_ is correspondingly underestimated by roughly 0.2 eV. In contrast, the valence band offset is in good agreement with reported experimental values and theoretical calculations.^[^ ^16^, ^24^, ^25^, ^26^ ^]^ For the conduction band offset, however, there has been little consensus on its accurate value. Estimations based on the electron affinity rule give an offset of roughly 0.3 eV.^[^ ^27^, ^28^ ^]^

### 2.2 Epitaxy of CdTe Shells

**Figure 2:**
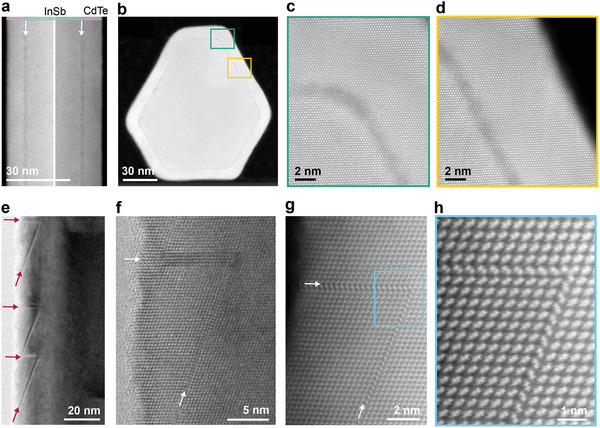
The impact of interfacial oxides. a) STEM images of the two opposing sides of the same nanowire imaged along the <112 > zone axis show a dark layer between the CdTe shell and the InSb core, indicated by the arrows. The dark layer is attributed to InSb oxides, appearing darker because they are of a lower electron density. b) A cross‐section of a nanowire imaged along the <111 > zone axis shows this oxide layer is all‐around, where high magnifications of the interface in (c) and (d) indicate that the limited thickness of this oxide layer still allows for epitaxy between InSb and CdTe. e) Despite epitaxy, defects in the CdTe shell are detectable (indicated by arrows) with high‐resolution transmission electron microscopy (TEM) along the <110 > zone axis. Two directions of planar defects are present, both parallel to {111} B planes: orthogonal to the long axis and at roughly 19° from the long axis. Different combinations of twinned layers in each direction occur. f) In the orthogonal direction four twinned layers are visible and in the inclined direction only a single pair of twin boundaries. g) Pairs of twin boundaries in each direction. HAADF‐STEM scans show that the defect starts at a specific point and then expands by two double {111} twin planes at a 71° angle. h) A high‐magnification scan of the starting point of the defect clearly reflects the interrupted crystal structure by the two twins.

The InSb nanowires studied here are grown using the vapor–liquid–solid technique on masked InSb (111) B substrates from gold catalysts defined by electron‐beam lithography, as detailed in,^[^ ^29^ ^]^ resulting in uniform arrays of nanowires (see Figure 1a). In our first approach to prevent the InSb nanowires from oxidizing, we try transferring them from the metal–organic vapor phase epitaxy (MOVPE)—where they are grown—to the molecular beam epitaxy (MBE) cluster—where the CdTe is deposited—under nitrogen overpressure. However, this nitrogen environment with low levels of oxygen ( <1 parts per million) is not enough to inhibit the InSb nanowires from oxidizing. The presence of these oxides manifests as a dark contrast layer between the InSb core and the CdTe shell in scanning transmission electron microscopy (STEM) imaging (**Figure** **2**a–d) and triggers the formation of defects (Figure 2e–h). The high defect density we observe in the CdTe shell of Figure 2e–h is consistent with reports on the presence of oxides on InSb surfaces affecting the quality of grown CdTe layers.^[^ ^30^ ^]^ The nanowire cross‐section of Figure 2b with a uniformly thick CdTe shell of 7 nm indicates that this (dark‐contrast) oxide layer at the core–shell interface is present all‐around the InSb nanowire. Yet, high‐magnification images of the interface close to a corner and parallel to a facet (Figure 2c,d) signify that the oxide layer is not fully formed as evidenced by the continuation of atomic columns locally from the core to the shell. In addition, the oxide layer is not thick enough to disrupt an epitaxial connection. Despite the limited thickness of this oxide layer, it is enough to trigger twin defects in the CdTe shell. As shown in Figure 2e–h, the twin defects are oriented parallel to {111} planes, that is perpendicular or inclined by ≈19° with respect to the long axis of the nanowire. In either direction, twinned layers come in pairs, one or multiples, such that the lattice orientation remains unchanged. Furthermore, high‐angle annular dark field (HAADF) STEM scans (Figure 2g,h) show that the defect expands from a given point by double {111} twin planes that form a 71° angle.

Accordingly, to prepare the InSb nanowires for defect‐free epitaxial CdTe shells it is essential to remove any surface oxides. In this work, the InSb nanowires are cleaned with atomic hydrogen in an MBE chamber prior to the growth of the CdTe shells. The reactive atomic hydrogen species are known to be effective in eliminating surface oxides from III–V semiconductors without changing stoichiometry or inducing roughness.^[^ ^31^, ^32^ ^]^ Other than preserving the pristine quality of the InSb nanowires, atomic hydrogen cleaning in an MBE system has the advantage of providing ultra‐high vacuum conditions throughout the entire process from cleaning to CdTe growth, thus ensuring that the nanowires remain oxide‐free after cleaning.

**Figure 3:**
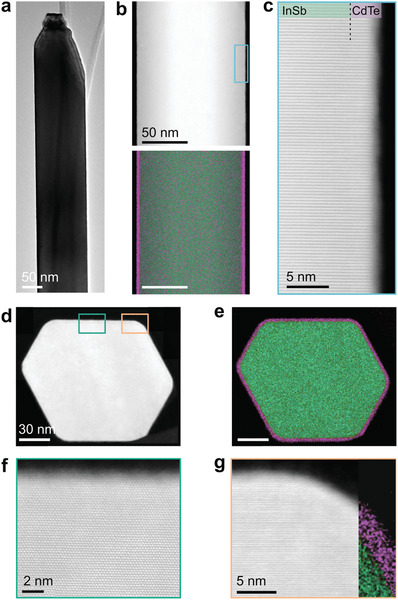
Growth of epitaxial uniform CdTe shells. a) A representative CdTe‐capped InSb nanowire imaged with TEM along the <112 > zone axis. b) A HAADF scan of a nanowire segment (<112 > zone axis) along with an energy dispersive X‐ray (EDX) map of a uniform CdTe shell (purple) around an InSb nanowire core (green). c) A zoom‐in on the framed region conveys epitaxy from core to shell, where the interface is virtually indistinguishable. d) A cross‐sectional HAADF scan accompanied by e) an EDX map shows a 2.5 nm full shell and two high magnification images, f) parallel to a facet and g) along the corner. Both reveal the abrupt interface and epitaxy between the InSb and the CdTe. In (g), an EDX map is overlaid to identify the CdTe and the InSb.

The parameters used for oxide removal with atomic hydrogen need to be carefully tuned, as the choice of parameters can be detrimental to the quality of both the InSb nanowires and the deposited CdTe shells. On the one hand, relatively low temperatures ( <200°C) are not enough to completely remove the native oxides, and thus lead to the growth of defected CdTe shells. On the other hand, higher temperatures ( >300 ° C) compromise the quality of the InSb nanowires by inducing surface roughness (see Figure S1, Supporting Information). Optimized cleaning parameters enable the growth of defect‐free epitaxial shells, free of interfacial oxides, as presented in **Figure** **3**. Details on atomic hydrogen cleaning are outlined in Section S2, Supporting Information.

Following the oxide removal, the CdTe is deposited from two separate cells, a Cd effusion cell and a Te cracker cell. During growth, the base pressure in the chamber is around 1 × 10^−9^ Torr. Three key considerations are necessary to suppress the formation of interface reactions which lead to Te‐rich interface layers. First, the nanowires are exposed to a Cd flux for 3 minutes prior to the introduction of Te. Second, growth proceeds under Cd‐rich conditions with a II/VI flux ratio = 3. We note that only a high II/VI flux ratio is not sufficient to hinder the formation of Te‐rich compounds at the core–shell interface. Particularly, shells deposited without pre‐exposure to Cd exhibit a Te‐rich interface layer, as measured with atom probe tomography (APT) (see Section S3, Supporting Information). These Cd‐rich conditions do not affect the shell stoichiometry because of the low sticking coefficient of Cd and its high vapor pressure—four orders of magnitude higher than Te.^[^ ^16^ ^]^ Third, relatively low growth temperatures are used (*T* ≈ 120–150°C) to ensure interdiffusion processes do not occur between the InSb and the deposited CdTe. Moreover, to promote the growth of smooth CdTe shells, low Cd and Te fluxes are used, yielding a growth rate of ≈0.002 monolayer s^−1^. Higher growth rates result in defected and rough shells. While higher temperatures enhance selectivity owing to a longer diffusion length of the adatoms—evident by the absence of CdTe deposition on the silicon nitride mask covering the nanowire substrate—they lead to thermal etch pits in both the InSb nanowires and substrate. The formation of these etch pits in InSb surfaces at elevated temperatures has been reported and can be minimized with an antimony overpressure.^[^ ^33^ ^]^ The thermal etch pits formed in the nanowires compromise the structural integrity of the nanowires causing them to bend (Figure S3, Supporting Information).

Accordingly, low growth temperatures and fluxes are used to drive the growth of defect‐free epitaxial CdTe shells. For instance, Figure 3a shows an overview bright field transmission electron microscopy (BFTEM) image of such an InSb–CdTe core–shell nanowire. The virtually indiscernible interface between the InSb and the CdTe in HAADF‐STEM imaging, in Figure 3b,c, relates not only to the absence of any interface layers but also to the similar atomic numbers of all the elements—indium, antimony, cadmium, and tellurium—composing both the core and shell. Accordingly, as presented in Figure 3b, EDX spectroscopy mapping is used to deduce the thickness of the grown shells. The thickness of this particular shell is 2.5 nm and is uniform along the length of the entire nanowire. Moreover, Figure 3c conveys the defect‐free epitaxy persisting from core to shell. Cross‐sectional studies of the nanowire allow for the investigation of the shell quality orthogonal to the long axis, as presented in Figure 3d–g. The accompanying EDX map illustrates the uniform CdTe shell thickness on all six facets. This uniform, full shell is enabled by rotating the substrate during the CdTe growth. High resolution imaging along the <111 > zone axis confirms the high‐quality and defect‐free epitaxy in the middle of a facet and at a corner. We emphasize that there is no visible contrast between the core and the shell demonstrating that there is abrupt epitaxy between InSb and CdTe without an interfacial layer.

**Figure 4:**
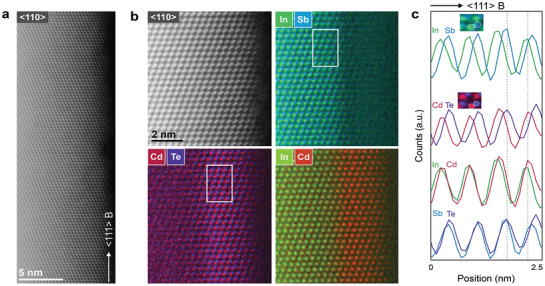
Atomic structure and composition of the CdTe shells. a) An InSb nanowire covered with a 2.5 nm CdTe shell imaged with HAADF‐STEM along the <110 > zone axis, as specified. b) A high magnification scan of (a) demonstrates the ABC stacking of the zinc blende crystal of both InSb and the epitaxial CdTe. Accompanying atomic resolution EDX maps reveal the positions of the elements composing the CdTe shell and InSb core along the <111 > B growth direction. c) Framed regions in (b) indicate along which areas the atomic profiles are taken. Integrating over the region yields the projection of the atomic positions on one line along the <111 > B. Inset are two high magnification images of the atomic resolution EDX maps, InSb and CdTe, to highlight the atomic positions of the elements in the core and shell. The atomic resolution EDX maps and the atomic profiles display the polarity of the shell with respect to the core, with Cd taking the position of In and correspondingly, Te that of Sb. This is further demonstrated by the absence of a shift in the line profiles of In (Sb) and Cd (Te), as highlighted by the vertical dashed lines.

This epitaxy is also clearly detectable in the high‐magnification scans taken along the <110 > zone axis of the HAADF scanning mode presented in **Figure** **4**a, where the zinc blende structure of both the InSb and the CdTe is recognizable. An atomic‐resolution composition mapping of the shell obtained with atomic‐resolution EDX in Figure 4b conveys the positions of the elements composing the core and the shell. These mappings reveal that the CdTe copies the polarity of the InSb nanowire. In particular, the (111) B layers orthogonal to the growth direction are terminated by antimony atoms, in line with the (111) B substrate orientation. Correspondingly, the (111) B planes of the shell are terminated by Te atoms and Cd takes the position of In (Figure 4b). This polarity is further demonstrated by atomic profiles taken along the InSb core and the CdTe shell where the projection of the atomic positions on one line along the <111 > B direction denotes the spatial overlap between In and Cd, and similarly Sb with Te in Figure 4c.

Close examination of the shells reveals that they do not oxidize (scans are taken at least 3 weeks after shell growth) evident by persistent atomic columns that are not bound by a low‐electron density material or amorphous structure. The inertness of CdTe stands in stark contrast to InSb, which readily oxidizes at very low levels of oxygen. The susceptibility of InSb to oxidation highlights the importance of these shells since they effectively hinder the formation of surface oxides around the InSb nanowires.

The structural quality of these shells is assessed for strain. As expected, because of the near lattice‐match, the shells are relaxed as confirmed by strain mapping provided in Figure S4, Supporting Information. A relaxed shell implies a large critical thickness, on the one hand, consistent with defect‐free shells for the range of studied thicknesses up to 15 nm. On the other hand, the nonstrained interface further confirms the absence of interface layers, since the commonly formed interfacial indium‐ and tellurium‐rich compounds have a lattice mismatch of roughly 5% with InSb.^[^ ^16^ ^]^

Tuning the CdTe shell thickness is simply achieved by varying the growth time, where a linear approximation of the growth rate yields roughly 5.4 nm h^−1^. We investigate shells with thicknesses from 2.5 up to 12 nm. We note that for shell thicknesses greater than 5 nm very slight roughness is observed with TEM along the <110 > zone axis (Figure S5, Supporting Information). Imaging the same shell along the <112 > zone axis does not reveal this roughness because images are taken parallel to a roughly 100‐nm‐long nanowire facet. Thus, aggregated nanoscale roughness is projected in the image plane. In contrast, <110 > zone axis viewing yields an image of the corner between two facets, thereby exposing any atomic scale roughness at the corners. This roughness shows up in projection at edges orthogonal to a <111 > direction. Although the exact topography and features of this roughness cannot be extracted, it could be related to the tendency of CdTe to form {111} facets with increased layer thickness, as described in [34, 35].

Eventually, growth is terminated by closing both the Cd and Te shutters, thus cool down proceeds without any fluxes. As a matter of fact, cooling down under a Te flux at low growth temperatures, for example, 120–150 °C, results in the deposition of Te‐rich CdTe globules on the nanowire facets as depicted in Figure S6, Supporting Information.

### 2.3 Electric Characterization of the InSb–CdTe Core–Shell Nanowires

**Figure 5:**
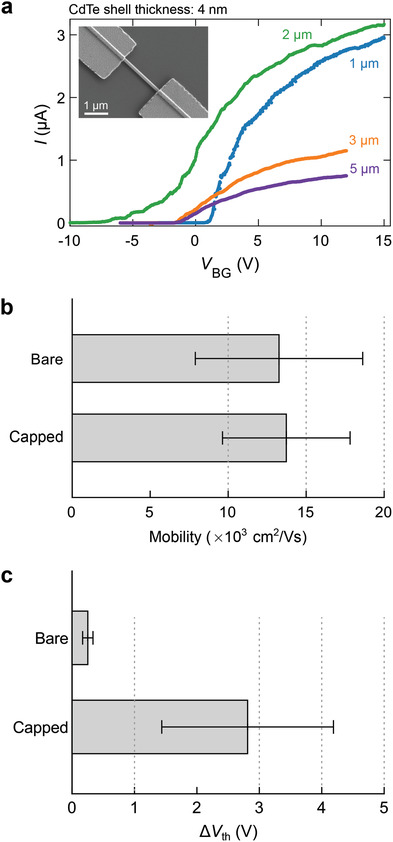
Mobility in CdTe‐capped nanowires. a) FET measurements at a bias voltage *V*_dc_ = 10 mV for four core–shell nanowires (CdTe thickness = 4 nm) with channel lengths *L* = 1, 2, 3, and 5 µm. Fitting these pinch‐off curves yields mobility μ = 21.5, 26, 14, and 17 × 10^3^ cm^2^ V^−1^ s^−1^ for 1, 2, 3, and 5 µm, respectively. Inset: SEM of a nanowire device. b) The average mobility for 4‐nm CdTe capped and bare InSb nanowires. Mobility is averaged for 1‐ and 2‐µm channel devices. c) Average hysteresis measured in 4‐nm CdTe capped and bare nanowires and is averaged for 1‐ and 2‐µm channel devices. Hysteresis is quantified by the threshold‐voltage difference Δ*V*_th_ between the forward and backward gate‐voltage sweeps.

The role of the epitaxial CdTe capping is evaluated by performing electron transport measurements on the core–shell nanowires. We note that we have not evaluated the tunnel barrier properties of the shell but studied the basic field‐effect transistor (FET) characteristics of the core–shell wires. While there are limitations to mobility extraction from FET measurements in nanowires which could result in inexact mobility values,^[^ ^36^, ^37^ ^]^ FET measurements remain a commonly used technique for characterizing nanowires.^[^ ^38^, ^39^, ^40^, ^41^ ^]^ We perform FET measurements at 4 K to extract the mobility μ. A typical device is presented in the inset of **Figure** **5**a. About 60 FET devices are fabricated with core–shell nanowire diameters of roughly 120 nm. Degenerate p‐doped silicon substrates covered with silicon oxide (SiO_2_) and hafnium oxide (HfO_*x*_) serve as a global back gate and titanium/gold contacts serve as the source–drain electrodes. Directly depositing the source‐drain contacts onto the CdTe shells, for the studied shell thicknesses of 4 to 12 nm, results in an open‐circuit, reflecting that these shells serve as good insulators. Therefore, prior to depositing the source‐drain electrodes, the CdTe is etched locally by argon milling to make contact to the conducting InSb core. Additional details on device fabrication are provided in Section S8, Supporting Information. The source–drain contact separations are *L* = 1, 2, 3, and 5 µm to ensure the long channel diffusive transport regime. Furthermore, these large separations ensure that any damage induced by the argon milling close to the source–drain contacts is eliminated and the measurements reflect the behavior of the segment between the contacts. Back‐gate voltage sweeps *I*(*V*_BG_) for the studied channel lengths and a fixed CdTe shell thickness of 4 nm are depicted in Figure 5a. Field‐effect mobility values are extracted from fits of the pinch‐off curves (Section S9, Supporting Information). The majority of the obtained mobility values are in the range of 1.0–2.7 × 10^4^ cm^2^ V^−1^ s^−1^ and no significant difference is found with uncapped, bare InSb nanowires (Figure 5b).

The equivalent mobilities for the CdTe shell and uncapped InSb nanowires indicate that the shell does not induce additional disorder to the InSb nanowires, thereby preserving the InSb nanowire properties. Possibly, thicker shells ( >12 nm) are required to passivate the nanowire surface and further confine the electrons in the nanowire, similar to InSb quantum wells, where relatively thick barrier layers ( >50 nm) are needed to achieve high mobility.^[^ ^42^ ^]^ Nevertheless, thicker shells would not be compatible with a tunnel barrier at the interface between a semiconducting nanowire and a superconductor, seeing as the tunneling probability decreases exponentially with barrier thickness.

While the mobilities are comparable for both CdTe‐capped and uncapped InSb nanowires, we observe a larger hysteresis between the forward and backward gate‐voltage sweeps for the CdTe‐capped wires compared to the uncapped wires, as shown in Figure 5c. The origin of this hysteresis is unknown, nevertheless it could be attributed to point‐defects, at the interface and in the CdTe shells, which are known to trap charges.^[^ ^43^, ^44^ ^]^ Point‐defects do not possess a lattice structure in any dimension and accordingly are not visible in our TEM studies of the CdTe shells. The relatively low substrate temperatures used during the CdTe growth could possibly lead to the formation of point defects. They could also be formed during the atomic hydrogen cleaning of the InSb surface, resulting in point‐defects at the InSb–CdTe interface. We note that pumping the device space for 96 h compared to 24 h can slightly reduce the hysteresis and lead to a slight increase in the fitted mobility (Figure S7, Supporting Information). However, this slight improvement indicates that the hysteresis is likely dominated by attributes inherent to the interface and the CdTe shells, for instance, adsorbates related to point‐defects and point‐defects.

## 3 Conclusion

We studied the InSb–CdTe material system in a core–shell nanowire configuration for potential applications in surface passivation and tunnel‐barrier devices. We determined the suitability of these heterostructures for the proposed applications based on the electronic structure of the InSb–CdTe interface and the quality of the CdTe epitaxy on the InSb cores. Importantly, the potential to use these CdTe shells in hybrid superconducting–semiconducting nanowire devices is proposed based on the obtained high‐quality InSb–CdTe interfaces and the small conduction band offset of this interface. In addition the comparable field‐effect mobilities for both uncapped and CdTe‐capped nanowires indicate the suitability of the CdTe shells to modulate the superconductor–semiconductor coupling without adding disorder to the device. We note that we would have expected with the type‐I band alignment of the InSb–CdTe interface, the nearly perfect epitaxy, and the high‐quality interfaces an improvement in electron mobility in the CdTe‐capped nanowires compared to the uncapped InSb wires. The similar mobility values in both uncapped and CdTe‐capped nanowires likely suggests that thicker CdTe shells (>12 nm) are required to confine the electron wavefunctions to the InSb core and attain higher mobility. While thicker shells are likely required for surface passivation, they are incompatible with tunnel‐barrier devices since the tunneling probability decreases exponentially as a function of barrier thickness. Both functionalities—surface passivation and tunnel barriers—can be combined in a single nanowire device by growing asymmetrically thick shells, where the part of CdTe shell that will be in contact with the metal or superconductor is thin and the remaining nanowire facets are covered by a thick CdTe shell. Such hybrid nanowire devices that would combine reduced disorder—improved mobility—with tunable superconductor‐semiconductor coupling could possibly open up an avenue for a new generation of topological nanowire devices.

## 4 Experimental Section

Experimental details are provided in the Supporting Information. Data that support figures, transport measurement plots, and other findings are available at https://doi.org/10.5281/zenodo.5592057.

## Conflict of Interest

The authors declare no conflict of interest.

## Supporting information

Supporting Information

## Data Availability

The data that support the findings of this study are openly available in Zenodo at https://doi.org/10.5281/zenodo.5592058, reference number 5592058.
