# Supplementary material for: Electronic Structure and Epitaxy of CdTe Shells on InSb Nanowires
Source: Adv Sci (Weinh). 2022 Feb 18;9(12):2105722. doi: 10.1002/advs.202105722 (PMC9036012; doi:10.1002/advs.202105722)
Supplement: Supplementary file 1 — Supporting Information [file ADVS-9-2105722-s001.pdf]

ADVANCED SCIENCE
Open Access

Supporting Information

for *Adv. Sci.*, DOI 10.1002/advs.202105722

Electronic Structure and Epitaxy of CdTe Shells on InSb Nanowires

*Ghada Badawy, Bomin Zhang, Tomáš Rauch, Jamo Momand, Sebastian Koelling, Jason Jung, Sasa Gazibegovic, Oussama Moutanabbir, Bart J. Kooi, Silvana Botti, Marcel A. Verheijen, Sergey M. Frolov and Erik P. A. M. Bakkers**

Supplementary Information

I. DENSITY FUNCTIONAL THEORY CALCULATIONS

All density functional theory (DFT) calculations are performed using the Vienna *ab initio* simulation package (VASP) [1]. These calculations employ the VASP implementation of the generalized Kohn-Sham scheme with the projector augmented-wave (PAW) method [2]. A plane-wave cutoff of 274.3 eV is used and spin orbit coupling is included in all calculations. While the results presented in the main text are obtained using the Heyd-Scuseria Ernzerhof (HSE06) hybrid exchange-correlation functional [3, 4], in this section results from additional exchange-correlation (xc) functionals are considered: the Perdew-Burke-Ernzerhof (PBE) [5], the modified Becke-Johnson (mBJ) [6], and the local mBJ (lmBJ) [7] (see Table I).

For the bulk calculations, the lattice constants of InSb and CdTe are set to the experimental values of 6.479 Å and 6.482 Å, respectively [8]. These calculations provide the bulk bandgaps (E_g) of both materials. For the supercell calculation, each material is represented by 10 monolayers, i.e., 40 atoms in total, connected at a non-polar (110) plane and the Brillouin zone is sampled using a Γ-centered 6 × 6 × 2 **k**-point grid. The tabulated valence and conduction band offsets —ΔE_v and ΔE_c, respectively—are extracted using both the individual bulk calculations and the supercell calculation [9]. In particular, from the supercell calculation, the potential offset, ΔV, between InSb and CdTe is obtained using a macroscopic average of the electrostatic potential. This value is then used to align the energy levels obtained from the two separate InSb and CdTe bulk calculations.

XC functional	E_g (InSb)	E_g (CdTe)	ΔE_v	ΔE_c
PBE	0.0	0.49	-0.63	-0.14
mBJ	0.24	1.57	-0.65	0.68
lmBJ	0.21	1.54	-0.68	0.65
HSE06	0.28	1.27	-0.84	0.15
experimental	0.24	1.45	-0.87	0.34*

Table I. **Bulk band structures and band offsets.** Values are given in electron Volts (eV). Experimental bandgaps are obtained from [10–12]. The valence band offset is estimated from x-ray photoelectron spectroscopy in [13]. The asterisk signifies that the experimental ΔE_c is calculated from the experimental bandgaps and ΔE_v.

As shown in Table I, the PBE functional predicts InSb to be a metal, i.e., with a zero bandgap, and thus this standard exchange-correlation functional cannot be used to describe the system. The mBJ, the lmBJ and, the HSE functionals give results which agree well with the experimental values. Specifically, HSE gives an accurate valence band offset compared to experiments, but it underestimates the bandgap of CdTe and accordingly ΔE_{c} by roughly 0.2 eV. Generally, the mBJ and the lmBJ exchange-correlation functionals are known to accurately describe bandgaps, while being numerically more feasible than hybrid functionals, which is also true for InSb and CdTe. However, both underestimate ΔE_{v} and correspondingly overestimate ΔE_{c} by approximately 0.3 eV, consistent with calculations in [14].

II. ATOMIC HYDROGEN CLEANING

Prior to the deposition of the CdTe shells, the native oxides surrounding the nanowires need to be removed to ensure epitaxy. Before introducing the nanowire chips into the MBE system, they are glued to a molybdenum holder next to a temperature-reference chip of gallium arsenide (GaAs). The holder is then degassed for two hours at 300° C to ensure the desorption of any water molecules or unwanted adsorbates. Once degassed, the holder is inserted in the cleaning chamber. Table II summarizes the relevant cleaning parameters. Once the oxide is removed, the nanowire chips are kept in the chamber until they have cooled down to 80° C and the chamber pressure has reached 3×10^{-9} Torr. The nanowire chips are then transferred through an ultra-high vacuum transfer tube to the growth chamber.

A series of cleaning times in combination with substrate temperatures have been investigated, as outlined in Figure S1. While too high temperatures ($>$ 300° C) induce roughness and damage the InSb nanowires surfaces, low temperatures are not sufficient to remove the native oxides. The remaining oxides manifest as a dark contrast at the InSb-CdTe interface in high-angle annular dark field (HAADF)

**Parameter**	**Value**
Substrate temperature	250° C
Hydrogen flow	20 sccm
Filament temperature	1200° C
Chamber pressure	2.5×10^{-5} Torr
Cleaning duration	45 minutes

Table II. **Atomic hydrogen cleaning parameters.** The substrate temperature is measured on the surface of the temperature-reference chip. During the entire cleaning procedure the holder is rotated. When idle, the chamber pressure is roughly 6×10^{-10} Torr.

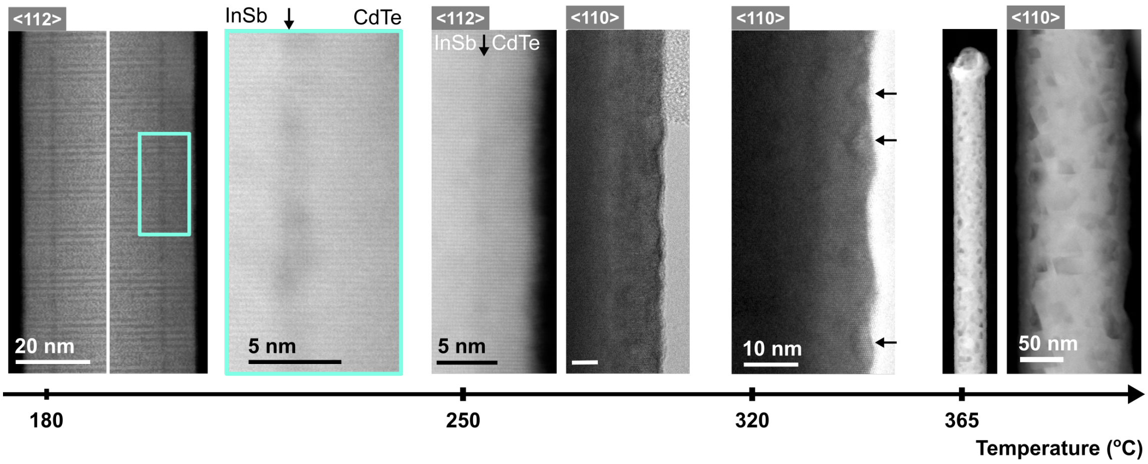

Figure S1. **Effect of substrate temperature during atomic hydrogen cleaning of core-shell nanowires.** Hydrogen cleaning of the InSb core at 180° C does not fully remove the oxide, as evident by the dark layer at the InSb-CdTe interface. A high magnification of the interface shows that this oxide layer is not of uniform thickness. At 250° C, this oxide layer is barely discernible and along the $< 110 >$ zone axis, the shell is defect-free signifying that the oxides are mostly removed. While 320° C instigates the onset of roughness in the InSb, at 365° C structural damage of the nanowire is detectable. In high-angle annular dark field imaging at both the $< 112 >$ and $< 110 >$ zone axes, damage manifests as pits on the nanowire surface.

imaging, reminiscent of a low electron-density material— compared to InSb and CdTe. Accordingly, higher temperatures and longer cleaning times have been used to completely get rid of the oxide, since the cleanliness of the InSb surface determines to a great extent the quality of the grown CdTe shells [15]. Although the defect density in the CdTe shells is greatly minimized with optimized atomic hydrogen cleaning temperature and duration, implying that the native oxides are successfully removed, a dark interface contrast, though subtle, persisted regardless of cleaning conditions (Figure S1: 250°). The persistence of this interface layer leads us to conclude it is possibly arsenic (As) originating from the GaAs temperature-reference chip. In particular, during atomic hydrogen exposure, the GaAs chip is also being cleaned and is possibly releasing As at these temperatures which is being redeposited on the InSb nanowires. To substantiate the presence of As at the InSb-CdTe, atom probe tomography studies are used to analyze the nanowires since this interface layer is not detectable with elemental dispersive x-ray (EDX) spectroscopy.

III. ATOM PROBE TOMOGRAPHY ANALYSIS

For the atom probe tomography studies, the InSb nanowires were cleaned using atomic hydrogen at a substrate temperature of 250° C for 20 minutes followed by the growth of a 50-nm CdTe shell. These shells were grown with a Cd/Te ratio of 3 and a Cd pre-exposure time of one minute. These cleaning and growth conditions yield results akin to the one shown in Figure S1: 250° with a very subtle dark

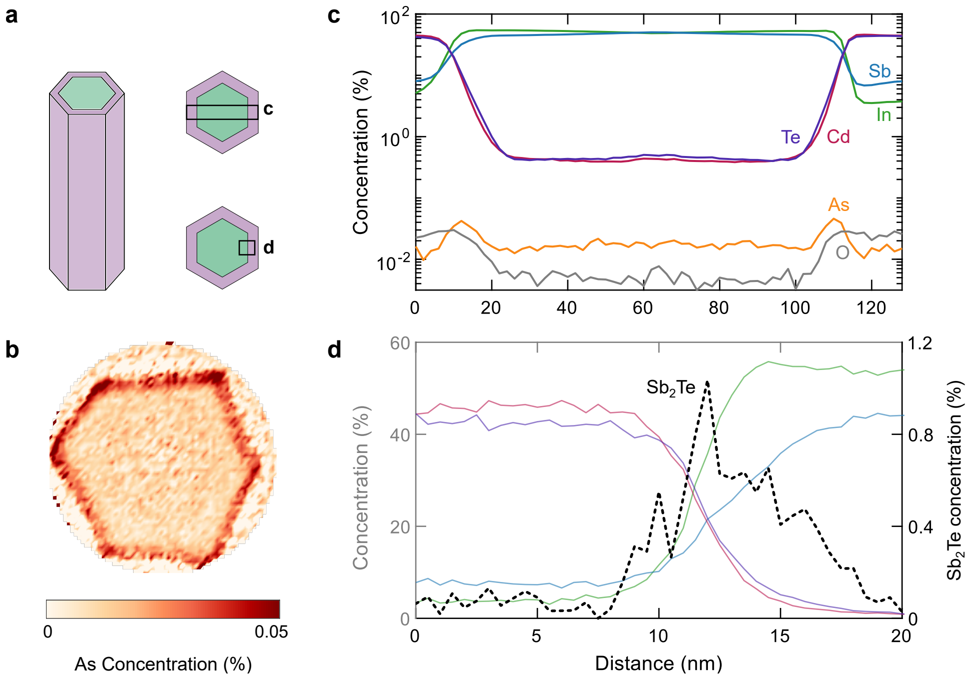

Figure S2. **Atom probe tomography analysis. a.** A schematic of an InSb core (green) and a CdTe shell (purple) and two top-view images showing the analyzed profiles in **c.** and **d.**, a rectangular profile along the entire diameter and an interface profile, respectively. **b.** Two-dimensional mapping of the arsenic concentration, reflecting a slight enrichment at the core-shell interface. **c.** This arsenic concentration is also visible along the rectangular profile with two peaks at the interface of about 0.04%. Roughly 0.03% of oxygen is also present at the interface. The overlapping regions between the InSb and the CdTe are not due to interdiffusion but just peak overlaps. Within the detection resolution of roughly 0.1-1% limited by these peak overlaps, there is no measurable interdiffusion. **d.** An interface profile spanning a 20-nm region shows that Sb and Te are clustering up at the interface, forming a 3-4 nm thick layer. This layer contains Sb_2Te ions created during atom probe tomography indicating a mixed interface region/layer that incorporates both Sb and Te atoms rather than an abrupt interface between InSb and CdTe.

contrast in high-angle annular dark field (HAADF) imaging. Accordingly, we use the results from the atom probe tomography to fine-tune the growth conditions and to understand the origin of this darker interface layer, such that we can eliminate it. The results from the atom probe tomography analysis are presented in Figure S2 and confirm the presence of As at the InSb-CdTe interface. Moreover, very low levels of oxygen (approximately 0.03%) are measured. Importantly, the dark interface layer is mostly attributed in this case to a tellurium-rich, possibly a Sb_2Te, layer. As shown in Figure S2d, this Sb_2Te concentration extends into the InSb, confirming the presence of a Te-rich interface region.

To address the arsenic issue, the GaAs temperature-reference chip is covered with a 50 nm silicon nitride mask. The mask hinders the release of arsenic from the GaAs surface and thereby minimizes arsenic re-deposition on the nanowire surfaces during atomic hydrogen cleaning. With regards to the

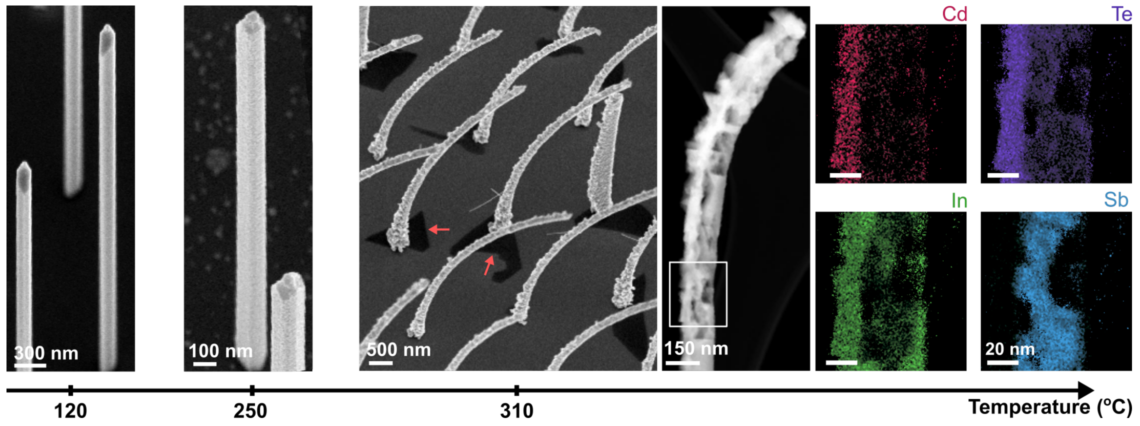

Figure S3. **CdTe shell growth at different temperatures.** Shell growth at 120° C yields smooth shells and a complete layer on the mask. At 250° C, roughness is already detectable within the resolution of the scanning electron microscope. Parasitic growth on the substrate reflects an increased diffusion length, resulting in islands rather than a complete layer. Even more roughness is induced at 310° C in addition to thermal etch pits in the substrate (tiny red arrows) and the nanowires are bent. A close examination of a single nanowire shows that the bending is instigated by pits in the nanowires. These voids are additionally visible in elemental dispersive x-ray mapping. The clustering of In towards the shell (overlapping with Te) signifies that possibly In_2Te_3 reactions took place at these temperatures.

oxygen levels, the cleaning time is extended from 20 minutes to 45 minutes to ensure the complete removal of the native oxides. As for the Te-rich interface layer, its formation is suppressed by flushing the growth chamber with Cd before introducing Te, as detailed in the main text. These optimizations in the cleaning and growth parameters yield clean, smooth and abrupt InSb-CdTe interfaces evidenced by epitaxial shells absent of dark-contrast interfacial layers as discussed in the main text.

IV. CDTE GROWTH

Substrate temperature

The growth of CdTe shells takes place at relatively low temperatures, e.g., 120° C, since higher temperatures ($\approx$ 250°) result in rough and defected shells (Figure S3) and are known to promote interface reactions between the InSb and the CdTe. Even higher temperatures ($>$ 300° C) compromise the structural integrity of the InSb nanowires. As shown in Figure S3, high temperatures instigate the liberation of Sb from the nanowires and the InSb substrate. This release of Sb manifests as pits in both the nanowires and the substrate, causing the nanowires to bend. These higher temperatures increase the adatom surface-diffusion length, evident by the absence of deposition on the masked substrate surface. Conversely, at the optimal growth temperature of 120° C, the substrate surface is covered with a CdTe layer.

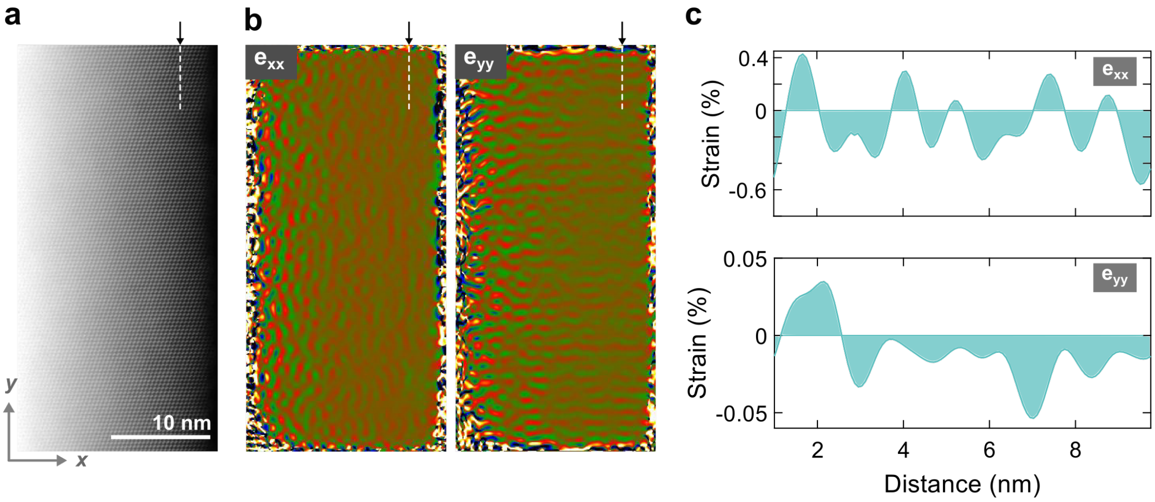

Figure S4. **Strain mapping. a.** HAADF-STEM image of an InSb-CdTe nanowire taken along the < 110 > zone axis with the interface indicated by an arrow. **b.** The strain mapping of lattice spacing differences along the x and y directions for the HAADF-STEM image shown in **a.** indicates a strain-free interface. **c.** Line profiles of the strain maps in **b.** show signal fluctuations below 0.5% from the average, thus confirming the absence of strain. The indiscernible InSb-CdTe interface in the strain maps further substantiates a relaxed and epitaxial interface.

V. STRAIN MAPPING

The InSb-CdTe core-shell nanowires are characterized to assess whether the interface is under strain. For this, an atomic resolution HAADF-STEM image is used where two $< -111 >$ reflections in the fast Fourier transform (FFT) diffraction patterns are selected. The strain maps along the x and y directions, the e_{xx} and e_{yy} images respectively, do not show any clear edges at the interface and the signal fluctuation is less than 0.5% from the average (Figure S4). There is thus no clear indication of strain, within the detection limit of this technique. The absence of strain is consistent with the almost perfect lattice match between InSb and CdTe.

VI. NANO-SCALE ROUGHNESS ALONG THE $< 110 >$ ZONE AXIS WITH INCREASING SHELL THICKNESS

Tuning the CdTe shell thickness is simply achieved by varying the growth time. For an increasing shell thicknesses (greater than 5 nm) very slight roughness is observed in the shell along the < 110 > zone axis with transmission electron microscopy (Figure S5). Imaging the same shell along the < 112 > zone axis does not reveal this roughness, since the nanowire is imaged parallel to a roughly 100 nm long nanowire facet, where a summation of nanoscale roughness is projected in the image plane. In contrast, in the < 110 > zone axis the nanowire is viewed at the corner between two facets, thereby

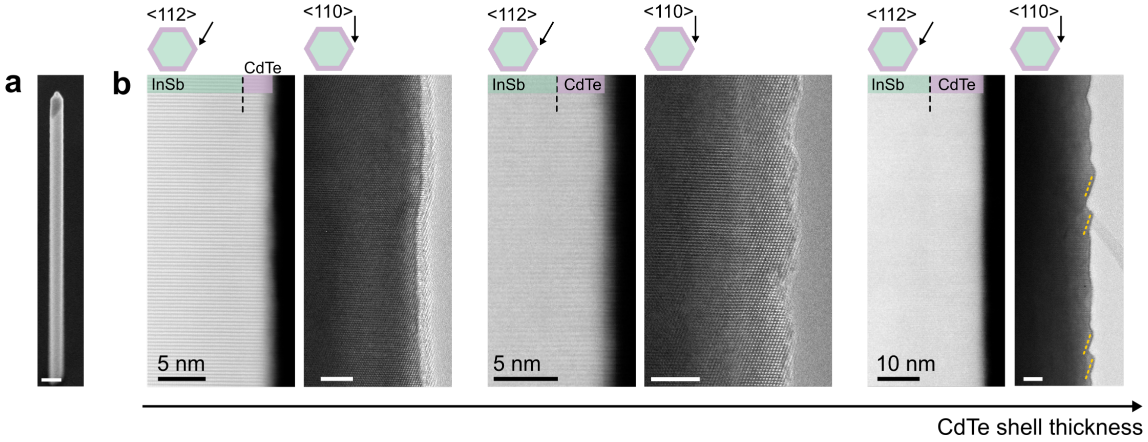

Figure S5. **Propensity to form nanofacets with increased CdTe thickness. a.** An SEM image of single InSb nanowire covered with a CdTe shell. Scale bar is 200 nm. **b.** HAADF scans and bright-field TEM images taken along the specified zone axes of nanowires with differently thick CdTe shells of 2.7 nm, 4 nm and 12.5 nm, respectively. Along the $< 112 >$ zone axis, the CdTe shells appear atomically flat. Along the $< 110 >$ direction, however, slight roughness is discernible and develops into well-defined non-vertical edges for the thickest shells. The roughly 13 nm shell is most likely terminated by $\{111\}$ planes, as indicated by the yellow dashed lines.

exposing any atomic scale roughness. This roughness shows up in projection at edges orthogonal to a $< 111 >$ direction. Although the exact topography and features of this roughness cannot be extracted, it could be due to an increased tendency of CdTe to form $\{111\}$ facets with increased layer thickness, as already disclosed [16, 17].

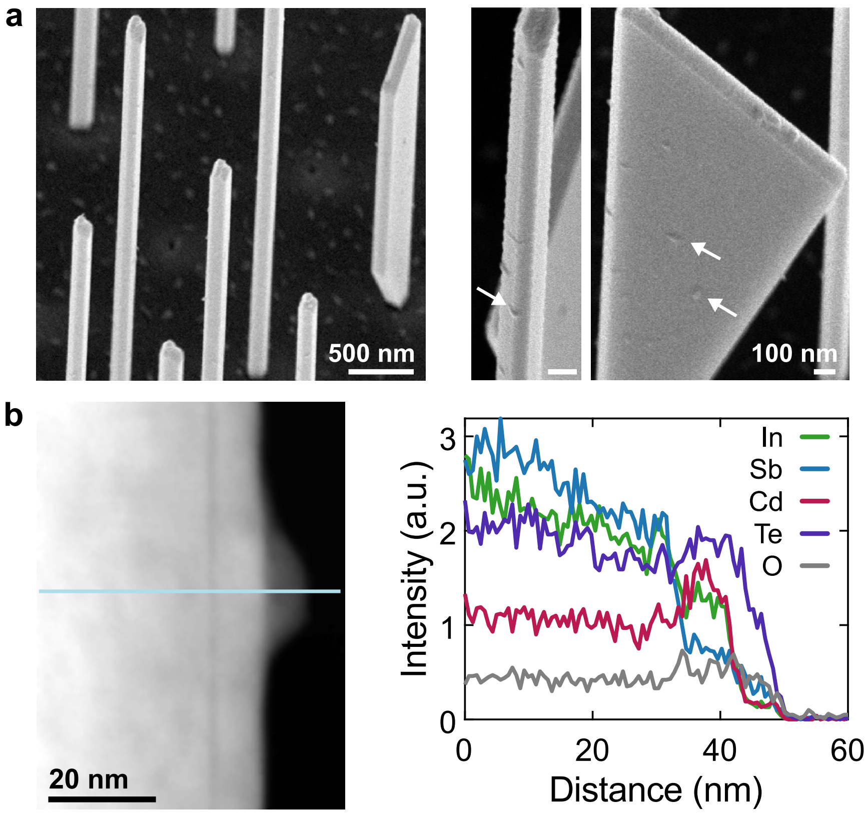

Figure S6. **Cool-down under Te flux.** **a.** SEM images taken at 30°-tilt showing tiny globules on the substrate surface and on top of the CdTe shells. Arrows point to representative globules on a nanowire and a nanoflake (scale bar: 100 nm). **b.** HAADF-STEM image displaying an area with a globule. An EDX line scan is acquired along the blue line. The composition profile indicates the globule has a higher Te content compared to the CdTe shell.

VII. COOL-DOWN UNDER TE FLUX

CdTe shell growth is terminated by closing both the Cd and Te shutters. However, for experiments in which a Te flux is supplied during cool-down of the substrate for 15 minutes, Te-rich CdTe globules are deposited on the CdTe shell (Figure S6). Conversely, for shells grown at higher temperatures (200° C) these globules are missing, likely related to a longer diffusion length.

VIII. DEVICE FABRICATION DETAILS

The fabrication process for mobility devices is described:

- p^{++}-doped Si substrates are covered by 285 nm SiO_x via dry oxidation and 15-nm hafnium oxide (HfO_x) via atomic layer deposition, onto which titanium/gold alignment markers are defined.

- The nanowires are deterministically transferred by a micro-manipulator from the growth chip onto the global back-gate chips. SEM images allow for designing the devices with respect to the alignment markers.

- The e-beam resist, PMMA 950 A4 is spun at 6000 rpm for 1 minute onto the chips, after which it is baked at 175° C for 15 minutes.

- The pattern of the contacts is defined by electron-beam lithography on the resist. Source and drain contacts are separated by InSb channels of lengths 1, 2, 3, and 5 μm.

- The chips are developed in a solution of MIBK:IPA (1:3) for 1 minute and in IPA for 1 minute.

- Prior to the metal deposition, the chips are cleaned for 30 seconds with a 200-Watt oxygen plasma and a flow of oxygen equal to 78 sccm.

- The substrate is loaded into an e-gun evaporator. The CdTe shell is removed via Ar milling set to 250 V and 15 mA for 3 minutes (for the 4-nm CdTe shell). This duration is divided into 30-second intervals to avoid over-heating of the nanowires.

- In the same chamber, a 10-nm layer of titanium followed by a 150-nm layer of gold are evaporated to form the contacts.

- The resist is lift-off in acetone at room temperature overnight.

IX. MOBILITY MEASUREMENTS

For the study of the field-effect mobility μ, nanowire field-effect transistor (FET) devices were fabricated as described in Section VIII. Based on the channel lengths L used, the diffusive long-channel regime is assumed. Hence, the current I as a function of back-gate voltage V_{BG} can be modeled by

$$I(V_{\mathrm{BG}}) = \frac{V_{\mathrm{dc}}}{(L^2/\mu C)(V_{\mathrm{BG}} - V_{\mathrm{th}})^{-1} + R_{\mathrm{c}}}, \tag{1}$$

where V_{dc} is the bias voltage. The saturation current is limited by the series resistance R_{c}, which includes the contact, filter, and line resistances. The current pinch-off is reached at the threshold voltage V_{th}. The value of the capacitance C is evaluated via a 3D Laplace solver for a typical nanowire device geometry, where the core-shell nanowire diameter is assumed to be 120-140 nm and the 15-nm hafnium oxide layer is accounted for. In this finite-elements model the InSb nanowire is treated as a metal [18]. The capacitance values for bare InSb nanowires and different CdTe shell thicknesses are given in Table III. All measurement data, logs, I-V curves, and codes used to extract the mobility are publicly available on Zenodo https://doi.org/10.5281/zenodo.5592057.

All FET nanowire devices for mobility extraction are measured in a dip-stick in helium at T = 4.2 K. Prior to cool down, the sample space is evacuated at room temperature for 24, 48 and 96 hours to efficiently desorb adsorbates from the nanowire surface. In Figure S7a-b, devices that have been pumped for 24 and 96 hours are presented to evaluate the effect of sample space evacuation on device performance. Particularly, a slight increase in mobility, going from 1.57×10^4 cm^2/Vs to 1.93×10^4 cm^2/Vs, is noted for the devices evacuated for 96 hours compared to 24 hours (Figure S7a). The data depicted in Figure S7a is for devices with a 4-nm CdTe shell. Longer sample space evacuation also reduces the measured hysteresis between the forward and backward back-gate voltage sweeps for the CdTe-capped wires, as illustrated in Figure S7b. The hysteresis is quantified by determining the difference in threshold voltages (ΔV_{th}) between both sweep directions. The devices studied in

	**Channel length**			
**CdTe thickness**	**1 μm**	**2 μm**	**3 μm**	**5 μm**
0 nm	36.8	85.8	134.8	232.8
2 nm	37.9	87.8	137.9	238.1
4 nm	37.2	86.6	136.0	235.1
7 nm	37.1	86.5	135.9	235.0
12 nm	36.6	85.6	134.8	233.1

Table III. **Capacitance values used for mobility fitting.** The device capacitance is evaluated for different channel lengths and CdTe shell thicknesses. Capacitance values are given in aF.

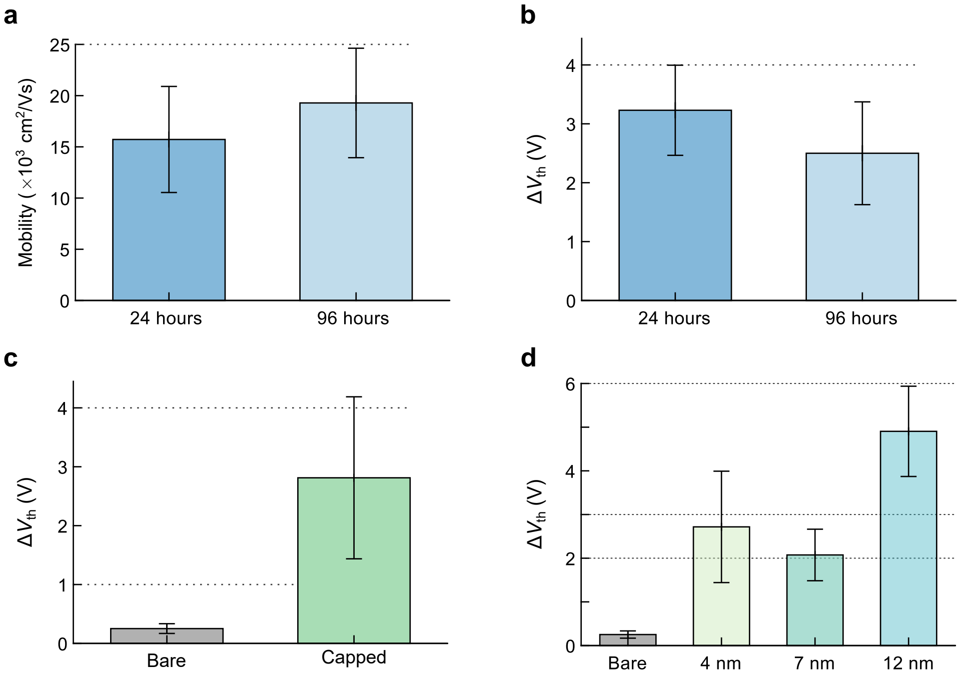

Figure S7. **Effect of sample space evacuation on mobility and hysteresis. a.** and **b.** FET nanowire devices with a 4-nm CdTe shell are evacuated for 24 and 96 hours. **a.** A longer evacuation leads to a slight mobility enhancement going from $1.57{\times}10^4$ cm^2/Vs to $1.93{\times}10^4$ cm^2/Vs. **b.** A decrease in hysteresis from $\Delta V_{\mathrm{th}} = 3.21$ V to 2.49 V, is noted for a 96-hour evacuation, compared to 24 hours. Hysteresis is quantified by the threshold-voltage difference ΔV_{th} between the forward and backward gate-voltage sweeps. **c.** CdTe-capped wires show a much larger hysteresis compared to uncapped, bare InSb nanowires. **d.** Hysteresis varies with CdTe shell thickness, with the largest hysteresis present for the 12-nm CdTe shell nanowires.

Figure S7b, indicate that on average hysteresis decreased from 3.21 V to 2.49 V as function of longer sample evacuation.

The presence of such a large hysteresis is associated with the CdTe shells, since hysteresis is nearly absent in uncapped, bare InSb nanowires (Figure S7c). Moreover, the slight improvement in the hysteresis for longer sample space evacuation suggests that something inherent to the shell dominates this hysteresis and is only partially caused by charges in the surrounding environment. While the origin of the hysteresis remains unknown, it is likely attributed to trapped charges within the shell (point defects in the CdTe shell), alternatively at one of the interfaces (the InSb-CdTe interface) or (CdTe-dielectric interface). We expect trapped charges in the CdTe shell to cause a larger hysteresis for thicker CdTe shells, as thicker shells would host more point defects. In contrast, trapped charges at the InSb-CdTe would be unaffected by the CdTe shell thickness, resulting in a similar hysteresis for all CdTe thicknesses. Trapped charges at the CdTe-dielectric interface are expected to result in a smaller hysteresis for increasing shell thickness, as charges are kept away from the InSb core for thicker

shells. While the results in Figure S7d show that the largest hysteresis exists for the thickest studied CdTe shells (12 nm) likely suggesting that the trapped charges are within the CdTe shells, this trend is not very conclusive. In large part, because we have more statistics on 4-nm CdTe shell nanowires compared to 7-nm and 12-nm shell wires. Accordingly, the shown trend is likely not representative.

References

[1] G. Kresse and J. Furthmüller, Efficient iterative schemes for ab initio total-energy calculations using a plane-wave basis set, Physical Review B **54**, 11169 (1996).

[2] P. E. Blöchl, Projector augmented-wave method, Physical Review B **50**, 17953 (1994).

[3] J. Heyd, G. E. Scuseria, and M. Ernzerhof, Hybrid functionals based on a screened coulomb potential, The Journal of Chemical Physics **118**, 8207 (2003).

[4] J. Heyd, G. E. Scuseria, and M. Ernzerhof, Erratum: "hybrid functionals based on a screened coulomb potential" [j. chem. phys. 118, 8207 (2003)], The Journal of Chemical Physics **124**, 219906 (2006).

[5] J. P. Perdew, K. Burke, and M. Ernzerhof, Generalized gradient approximation made simple, Physical Review Letters **77**, 3865 (1996).

[6] F. Tran and P. Blaha, Accurate band gaps of semiconductors and insulators with a semilocal exchange-correlation potential, Physical Review Letters **102**, 226401 (2009).

[7] T. Rauch, M. A. Marques, and S. Botti, Local modified becke-johnson exchange-correlation potential for interfaces, surfaces, and two-dimensional materials, Journal of Chemical Theory and Computation **16**, 2654 (2020).

[8] R. Farrow, G. Jones, G. Williams, and I. Young, Molecular beam epitaxial growth of high structural perfection, heteroepitaxial cdte films on insb (001), Applied Physics Letters **39**, 954 (1981).

[9] P. Borlido, M. A. Marques, and S. Botti, Local hybrid density functional for interfaces, Journal of Chemical Theory and Computation **14**, 939 (2018).

[10] I. Vurgaftman, J. á. Meyer, and L. á. Ram-Mohan, Band parameters for iii–v compound semiconductors and their alloys, Journal of Applied Physics **89**, 5815 (2001).

[11] T. L. Chu and S. S. Chu, Recent progress in thin-film cadmium telluride solar cells, Progress in Photovoltaics: Research and Applications **1**, 31 (1993).

[12] S. Greene, J. Singleton, T. Golding, M. Pepper, C. Langerak, and J. Dinan, Fundamental properties of high mobility insb-cdte heterojunctions, Surface Science **228**, 542 (1990).

[13] K. Mackey, P. Allen, W. Herrenden-Harker, R. Williams, C. Whitehouse, and G. Williams, Chemical and electronic structure of insb-cdte interfaces, Applied Physics Letters **49**, 354 (1986).

[14] Y. Hinuma, A. Grüneis, G. Kresse, and F. Oba, Band alignment of semiconductors from density-functional theory and many-body perturbation theory, Physical Review B **90**, 155405 (2014).

[15] S. Wood, J. Greggi Jr, R. Farrow, W. Takei, F. Shirland, and A. Noreika, Microstructural studies of cdte and insb films grown by molecular beam epitaxy, Journal of Applied Physics **55**, 4225 (1984).

[16] S. Neretina, Q. Zhang, R. Hughes, J. Britten, N. Sochinskii, J. Preston, and P. Mascher, The role of lattice mismatch in the deposition of cdte thin films, Journal of Electronic Materials **35**, 1224 (2006).

[17] I. Ribeiro, J. Suela, J. Oliveira, S. Ferreira, and P. Motisuke, Low temperature growth of high quality cdte polycrystalline layers, Journal of Physics D: Applied Physics **40**, 4610 (2007).

[18] Ö. Gül, D. J. Van Woerkom, I. van Weperen, D. Car, S. R. Plissard, E. P. Bakkers, and L. P. Kouwenhoven, Towards high mobility insb nanowire devices, Nanotechnology **26**, 215202 (2015).
